# Effect of His-Tag on Expression, Purification, and Structure of Zinc Finger Protein, ZNF191(243-368)

**DOI:** 10.1155/2016/8206854

**Published:** 2016-07-20

**Authors:** Dongxin Zhao, Zhongxian Huang

**Affiliations:** ^1^School of Chemistry and Chemical Engineering, Henan University of Technology, Zhengzhou 450001, China; ^2^Department of Chemistry, Fudan University, Shanghai 200433, China

## Abstract

Zinc finger proteins are associated with hereditary diseases and cancers. To obtain an adequate amount of zinc finger proteins for studying their properties, structure, and functions, many protein expression systems are used. ZNF191(243-368) is a zinc finger protein and can be fused with His-tag to generate fusion proteins such as His_6_-ZNF191(243-368) and ZNF191(243-368)-His_8_. The purification of His-tag protein using Ni-NTA resin can overcome the difficulty of ZNF191(243-368) separation caused by inclusion body formation. The influences of His-tag on ZNF191(243-368) properties and structure were investigated using spectrographic techniques and hydrolase experiment. Our findings suggest that insertion of a His-tag at the N-terminal or C-terminal end of ZNF191(243-368) has different effects on the protein. Therefore, an expression system should be considered based on the properties and structure of the protein. Furthermore, the hydrolase activity of ZNF191(243-368)-His_8_ has provided new insights into the design of biological functional molecules.

## 1. Introduction

Protein properties, structure, and functions are important considerations for the expression of proteins* in vitro*. Although proteins can be expressed* in vitro using* various expression systems, the formation of inclusion body, toxicity of exogenous proteins, modification of side-chain, and so forth all influence protein expression and can complicate the process of protein purification. Therefore, it has been difficult to express enough proteins for studying them* in vitro*. This also presents challenges for studying on ZNF191 and ZNF191(243-368).

Fusion expression systems (e.g., GST and His-tag) can facilitate protein expression and simplify protein purification [1, 2]. However, each expression system has unique characteristics that affect how it can be used. For example, if the target protein contains a thrombin digestion site, it cannot be easily cut from the GST fusion protein and cannot be expressed using the GST system. The His-tag expression systems are widely used because His-tags have a low molecular weight and do not affect protein structure and functions. This means that it is not necessary to separate the His-tag from the target protein [3, 4]. Moreover, His-tag fusion proteins can easily be purified by Ni-NTA affinity resin. Zinc finger proteins have been expressed, separated, and purified using the His-tag/Ni-NTA system [4–6]. However, the use of His-tag fusion proteins remains controversial [7, 8].

We established two His-tag expression systems for ZNF191(243-368). His_6_-tag was introduced at the N-terminal end and His_8_-tag was introduced at the C-terminal end of ZNF191(243-368). Ni-NTA resin was used for protein purification, which overcame the difficulty of ZNF191(243-368) separation and purification caused by the formation of inclusion body. The effects of the His-tag on ZNF191(243-368) properties and structure were evaluated using spectrographic techniques and hydrolase activity experiment. Our findings have furthered our understanding of the structure and folding processes of the zinc finger protein.

## 2. Materials and Methods

### 2.1. Materials

Restriction enzymes,* BamH* I,* Hind *III,* Nde* I, and* Xho* I, and T4 DNA ligase were purchased from New England Biolabs. Pfu DNA polymerase, dNTPs, isopropyl *β*-D-thiogalactoside (IPTG), Triton X-100, imidazole, ampicillin, and kanamycin were purchased from Sangon (Shanghai, China). Ni-NTA resin was purchased from Qiagen. All other reagents were of analytical grade. The pET-41b plasmid and* Escherichia coli* BL21(DE3) strain, pQE30 plasmid, and M15 strain (Novagen) were used as expression vectors and host strains. The pGEX-B plasmid was constructed in our lab. YM-5 ultrafiltration membrane was from Amicon. Sephadex G-25 was purchased from Pharmacia Biotech. Low- and mid-range protein markers were obtained from Bio Basic Inc.

### 2.2. Construction of Recombinant Plasmids

The pQE-ZF plasmid was constructed as previously described for expressing His_6_-ZNF191(243-368) [9]. The primers used to construct the ZNF191(243-368)-His_8_ expression system were as follows: up-primer: 5′-GGAATTCCATATGAGAAATCCCTCTCGAAAGAAACA-3′; down-primer: 5′-CCGCTCGAGAACTTCCACAACATTCAGAAG-3′. These primers were used to amplify ZNF191(243-368) from the pTSA-18 plasmid. Each segment was digested using* Nde* I and* Xho* I endonucleases and inserted into pET-41b vector. The resulting vector was named pET-ZF and transformed into* E. Coli* BL21(DE3) strain for expression.

### 2.3. Expression and Purification of His-Tagged ZNF191(243-368)

Plasmids pQE-ZF and pET-ZF were transformed into* E. coli* M15 and* E. coli* BL21(DE3) host bacteria, respectively. For His-tag expression systems, 3 mL of LB medium containing appropriate antibiotics (for the His_6_-tag expression system, ampicillin and kanamycin were added; for His_8_-tag expression system, kanamycin was added) was inoculated with a freshly isolated bacterial colony of the host strain carrying a recombinant vector. The medium was incubated overnight at 37°C and diluted to 1 : 100 in 3 mL of LB medium containing antibiotics with shaking until OD_600_ = 0.6; then IPTG was added at different concentrations and cultures were grown at different temperatures for different time. Cells of 1 mL medium grown and induced under different conditions were centrifugated and resuspended in 1 mL of lysate buffer, then mixed with 1 mL of 2 × SDS loading buffer, and boiled for 10 min, so the samples of whole cells were prepared. Then all samples were run on 15% SDS-PAGE gel and visualized by Coomassie Brilliant Blue R-250 staining. The target proteins were detected by comparison with protein standard markers, and the optimum conditions for* in vitro* expression were determined.

High expression of proteins was induced under optimum conditions. The expression and purification of ZNF191(243-368)-His_8_ were similar to those of His_6_-ZNF191(243-368) [9]. The cells harvested from 500 mL of LB medium grown and induced under the optimum conditions were suspended in 10 volumes of cell lysis buffer (50 mmol·L^−1^ NaH_2_PO_4_, pH 8.0, 300 mmol·L^−1^ NaCl, and 5 mmol·L^−1^ imidazole). And cells were lysed by lysozyme for about 1 h at 4°C, then treated with 5 U/mL DNase, and stirred for 30 min to degenerate nucleic acids at 4°C. Soluble and insoluble cell fractions were separated by centrifugation at 15,000 r/min for 30 min. Supernatants were mixed with Ni-NTA resin to purify fusion proteins according to manufacturer's manual. His-tagged proteins were eluted in the elution containing 50 mmol·L^−1^ NaH_2_PO_4_, 300 mmol·L^−1^ NaCl, and 250 mmol·L^−1^ imidazole, at pH 8.0. The eluted solution was concentrated using Amicon YM-5 and then passed through a Sephadex G-75 column to get rid of impurities and a Sephadex G-25 column to remove salts; then collected protein solution was lyophilized. The purified proteins were mixed with 2 × SDS loading buffer and boiled for 10 min to prepare the samples. The samples were detected by 15% SDS-PAGE gel.

### 2.4. UV-Vis Absorption Spectroscopy

The UV spectra of proteins were recorded on a HP 8453 Diode Array spectrophotometer (USA). Protein concentrations in 10 mM Tris-HCl solution (pH 7.5) were determined using Bradford's method. The concentration was 1 *μ*mol·L^−1^.

### 2.5. Circular Dichroism (CD) Spectroscopy

CD spectra of proteins were recorded between 190 and 250 nm using a J720 Jasco spectropolarimeter. The optical path length was 10 mm, and the concentration of protein solution with 10 mM Tris-HCl (pH 7.5) was 1 *μ*mol·L^−1^. The recordings were conducted at 25°C.

### 2.6. Zinc Ion Titration Test

ZNF191(243-368) and ZNF191(243-368) with His-tag were acidified and filtered through a Sephadex G-25 column to obtain proteins without zinc ions. CD curves of the zinc-free protein titrated by zinc ions were tested.

### 2.7. Urea Degeneration Test

A series of buffer solutions with different final urea concentrations (0, 1, 2, 3, 4, 5, 6, 7, and 8 mol·L^−1^) were prepared. The same protein amounts were added to all buffer solutions, which were let to stand for 10 min at room temperature to measure fluorescence.

### 2.8. Hydrolase Activity Test

Different amounts of protein solutions were added to the plasmid solution to give a final protein concentration of 1 *μ*mol·L^−1^. They were tested by 1% agarose gel electrophoresis after 24 h at 37°C.

## 3. Results and Discussion

### 3.1. Construction of Recombinant Plasmids

Plasmid pQE-30 has six continuous His codes at the N-terminal end of the exogenous gene and has ampicillin resistance.* BamH* I and* Hind* III digestion sites were used to clone the ZNF191(243-368) gene into the pQE-30 plasmid, producing the plasmid pQE-ZF. A RGS-His_6_ code is present behind the initiation codon of pQE-ZF, and the amino acid residues Gly and Ser are encoded by the* BamH* I site following the His-tag; therefore protein expressed by pQE-ZF contains eleven additional amino acid residues (RGSHHHHHHGS) at the N-terminal end of ZNF191(243-368). The expressed protein is called His_6_-ZNF191(243-368), and it has a molecular weight 1267 Da higher than that of ZNF191(243-368). In the plasmid pET-41b, the target gene was cloned between the* Nde* I and* Xho* I sites, generating a recombinant plasmid pET-ZF. Since the recognition site of* Nde* I has an initiation codon, no additional amino acid residues are present at the N-terminal end of ZNF191(243-368). However, the amino acid residues Leu and Glu are encoded by the recognition site of* Xho* I before eight continuous histidines; therefore an additional ten amino acid residues (LEHHHHHHHH) were detected at the C-terminal end of the target protein. The expressed protein is ZNF191(243-368)-His_8_ and has a molecular weight 1339 Da higher than ZNF191(243-368).

### 3.2. Expression and Purification of Proteins

The proteins were expressed under optimum conditions: the bacteria were grown to OD_600_ = 0.6 at 37°C and then induced by 0.1 mM IPTG for 6 h. His-tagged proteins were purified using Ni-NTA resin and detected by SDS-PAGE.

**Figure 1:**
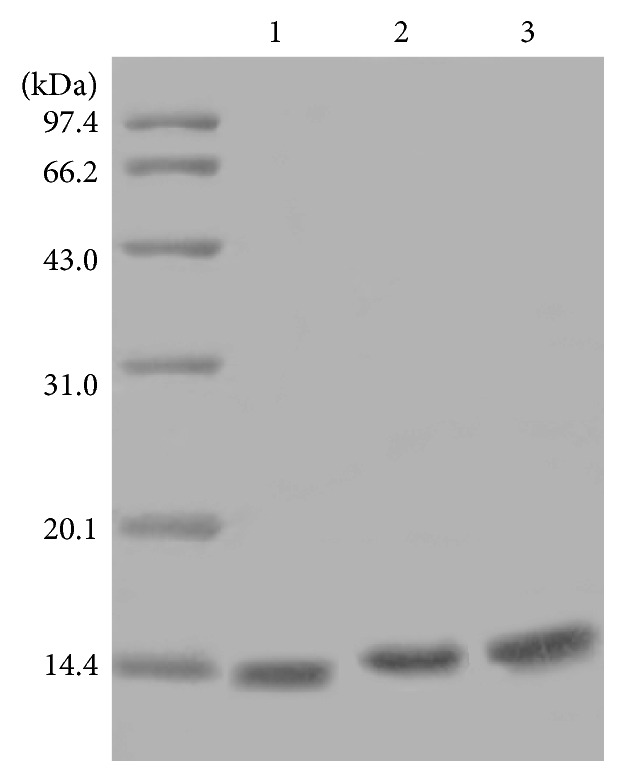
SDS-PAGE of zinc finger proteins. Lane 1: ZNF191(243-368), lane 2: His_6_-ZNF191(243-368), and lane 3: ZNF191(243-368)-His_8_.

Although the molecular weights of His_6_-ZNF191(243-368) and ZNF191(243-368)-His_8_ are higher than that of ZNF191(243-368), the difference between molecular weights was not observable by SDS-PAGE. The corresponding proteins showed a single band (Figure 1), indicating that the protein purity was higher than 90% and the position of the electrophoretic band was consistent with the expected molecular weight of the proteins. This indicated that target proteins were obtained* in vitro*, and His-tag expression systems were suitable for expressing and purifying ZNF191(243-368).

### 3.3. UV Spectra of Proteins

**Figure 2:**
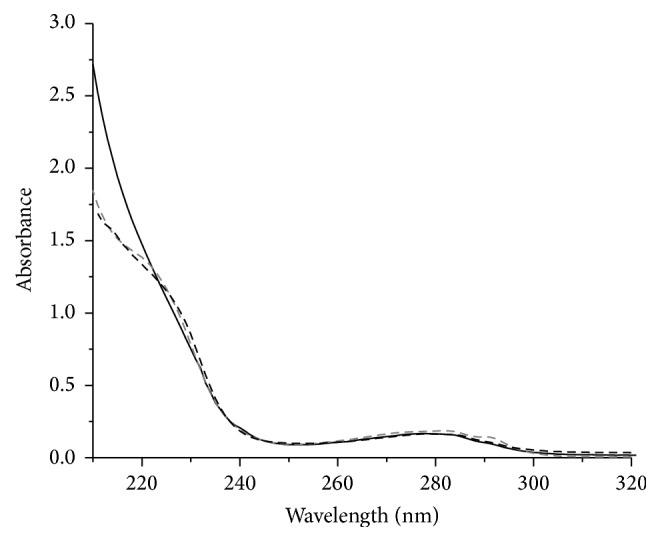
UV spectra of zinc finger proteins. Dark dashed line is ZNF191(243-368), gray dashed line is His_6_-ZNF191(243-368), and dark solid line is ZNF191(243-368)-His_8_.

In UV spectra of the three proteins (Figure 2), no significant difference was observed. All have absorption peaks of Phe and Tyr at 260–280 nm as well as the shoulder peak of the Zn-S bond formed by cysteine-Zn^2+^ coordinating at 230 nm, indicating that these proteins likely have similar structures [10, 11].

### 3.4. CD Spectra of Proteins

**Figure 3:**
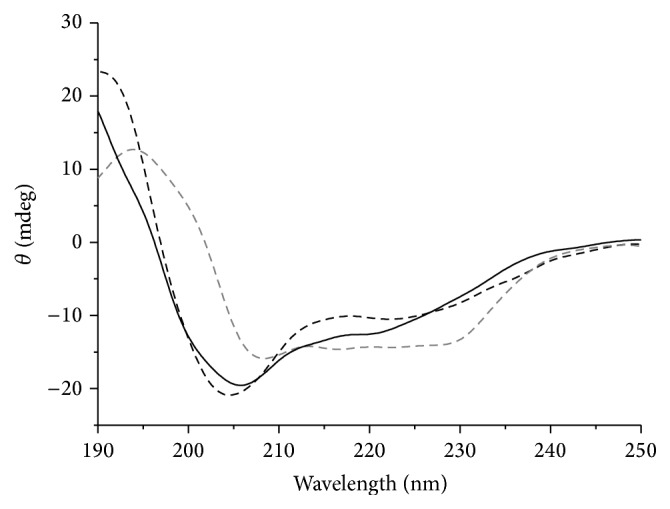
CD spectra of zinc finger proteins. Dark dashed line is ZNF191(243-368), gray dashed line is His_6_-ZNF191(243-368), and dark solid line is ZNF191(243-368)-His_8_.

According to the CD spectra of proteins in the far UV region (Figure 3), ZNF191(243-368) shows typical negative absorption peaks of an *α*-helix at about 205 nm and 222 nm as well as a positive absorption peak at about 190 nm [12]. The positive absorption peak shifted to 195 nm, and three shoulder peaks of negative peak at 210–230 nm in the CD spectrum of His_6_-ZNF191(243-368) indicated that His_6_-ZNF191(243-368) had more *β*-folding [13]. In addition, the similarity between the CD spectra of ZNF191(243-368)-His_8_ and ZNF191(243-368) showed that they had a similar structure. This indicated that the His-tags at the C-terminal end and N- terminal end of the zinc finger protein ZNF191(243-368) have different effects.

### 3.5. Zinc Ion Titration Test

Coordination of zinc ions with Cys and His in zinc finger peptides to form a tetrahedral molecule is necessary for maintaining the structure of the zinc finger domain. The CD spectrum is an effective means to test the folding of the zinc finger depending on the zinc ion coordination. The structure of the zinc finger domain is destroyed by dezincification and can be recovered by the addition of zinc ions; therefore zinc ions facilitate folding of the zinc finger peptide chain.

**Figure 4:**
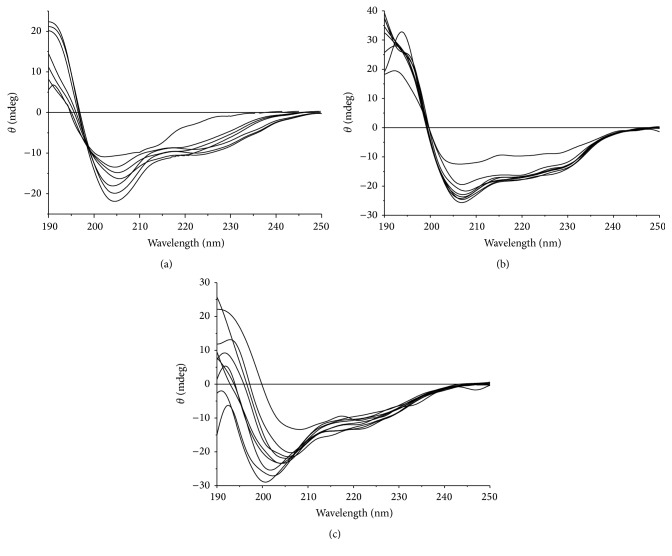
CD spectra of zinc finger proteins titrated by Zn^2+^. (a) ZNF191(243-368), (b) His_6_-ZNF191(243-368), and (c) ZNF191(243-368)-His_8_. The concentration of Zn^2+^ is from 0 (up) to 10 *μ*mol·L^−1^ (down).

The CD spectra of zinc-free protein titrated with zinc ions are shown in Figure 4. These show that zinc-free zinc finger proteins have a positive peak at about 190 nm and a negative peak at 205–235 nm, demonstrating that zinc-free zinc finger proteins still maintain some secondary structure and do not completely change into an irregular curve [14]. The addition of zinc ions returns the CD spectra of zinc-free ZNF191(243-368) and zinc-free His_6_-ZNF191(243-368) to those of proteins before dezincification shown in Figure 3. This indicates that their structure can be recovered by the addition of zinc ions [15]. In the zinc ion titration curve of zinc-free ZNF191(243-368)-His_8_, the negative peak of an *α*-helix at about 208 nm shifted to 200 nm, which is similar to the negative peak of the irregular curve at about 200 nm, indicating that ZNF191(243-368)-His_8_ does not have the original structure with zinc ion coordination because eight continuous histidines at the C-terminal end hinder the normal coordination of peptide chain with zinc ions.

Viewed from the perspective of inorganic chemistry, two ligands at the carboxyl end of one domain and two ligands at the amino end of the next domain may coordinate with one zinc ion in a protein containing continuous C_2_H_2_ zinc finger domains. Furthermore, two peptide chains may coordinate with one zinc ion [16]. The difference in the CD spectra of ZNF191(243-368)-His_8_ whether before or after zinc titration is caused by metal mismatching of His-tag at the C-terminal end. Of course, these are only speculations based on CD spectra. We hope to determine the structures of these proteins in the future through NMR and their crystal structure.

### 3.6. Urea Degeneration Test

**Figure 5:**
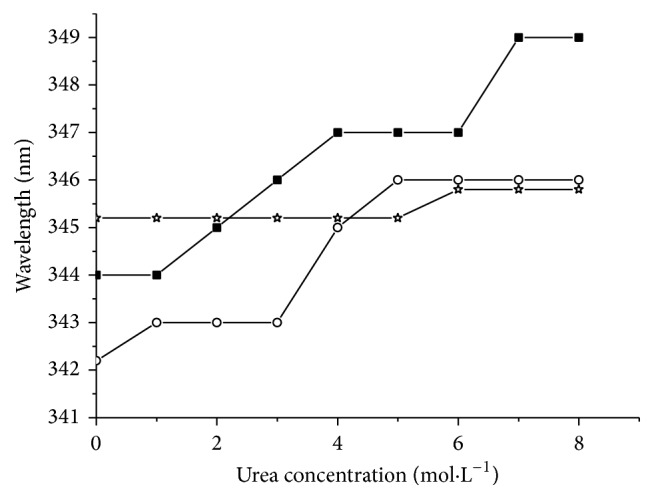
Urea denaturation-emission wavelength maximum versus urea concentration. ■ is ZNF191(243-368), ○ is His_6_-ZNF191(243-368), and ☆ is ZNF191(243-368)-His_8_.

ZNF191(243-368) contains four tyrosines. After urea degeneration, the microenvironment of tyrosines in proteins will be changed, so the fluorescence spectra of proteins will be varied accordingly to manifest changes of the protein structure [17, 18]. Displacement variations of the maximum emission wavelength of different zinc finger proteins under different urea concentrations are shown in Figure 5. The unfolding of proteins under different urea concentrations was analyzed based on the fluorescence spectra.

Under low urea concentrations, the maximum emission peak wavelength of the fluorescence spectrum of ZNF191(243-368) was about 344 nm, which shifted to 350 nm with increasing urea concentration. The maximum emission peak wavelength of His_6_-ZNF191(243-368) shifted from 342 to 346 nm. The maximum emission peak wavelength of the fluorescence spectrum of ZNF191(243-368) and His_6_-ZNF191(243-368) had the same red shift, suggesting that tyrosine residues in the protein moved from the original nonpolar environment in folding proteins to the polar environment in unfolding proteins. This was in accordance with the structure of zinc finger proteins. Since tyrosine in ZNF191(243-368) is the key amino acid residue of the hydrophobic core in the classical “finger-like” structure, it is exposed in solvent gradually during protein unfolding [19]. However, the maximum wavelength of ZNF191(243-368)-His_8_ did not change as the urea concentration increased, implying that the environment of tyrosine was changed slightly during unfolding. Therefore, His-tags at the N-terminal end and C-terminal end of proteins influence the structure of ZNF191(243-368) in different ways. Whether this difference influences protein functions remains to be elucidated.

### 3.7. Hydrolase Activity Test

**Figure 6:**
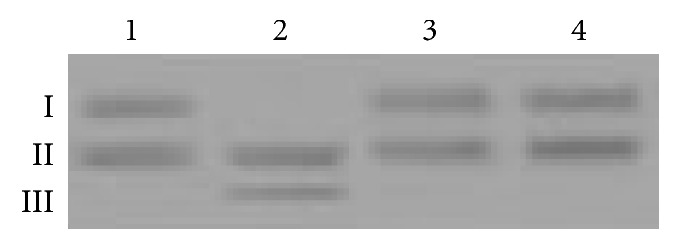
Cleavage of the plasmid DNA by zinc finger proteins. Lane 1 is pGEX-B not containing proteins, lane 2 is pGEX-B containing ZNF191(243-368)-His_8_, lane 3 is pGEX-B containing His_6_-ZNF191(243-368), and lane 4 is pGEX-B containing ZNF191(243-368).

The special hydrolysis of the phosphate ester linkage in DNA by natural nucleases has been a problem. Artificial design of hydrolytic nucleases will contribute more identifiable DNA sequences, which will benefit medicine design and gene therapy. At present, most design strategies are to connect DNA binding elements and DNA lipolytic elements or build up an active site of hydrolase in DNA binding peptides [20–22]. Many zinc finger proteins recognize specific DNA sequences. New zinc finger proteins have been designed to recognize unique DNA sequences and extend the recognition length of DNA [23–25]. It would be useful if a nuclease with a zinc finger structure could be designed, because the recognized site of zinc finger domain is not a symmetric sequence. The Cys in SP1 was mutated to His to successfully develop a zinc finger peptide with DNA hydrolysis function [26]. This reminds us of whether hydrolytic activity will be introduced into the zinc finger peptide or zinc finger protein through His-tag. Here, we chose plasmid pGEX-B containing a GGAGGG site to study the hydrolytic activity of His-tagged proteins. The pGEX-B plasmid was obtained from the DNA binding experiment of ZNF191(243-368) [27], and the electrophoresis results were shown in Figure 6. The plasmids all had two main bands.

Generally, extracted plasmids have two, sometimes three, electrophoretic bands. The plasmid at superhelix state (I) moved the quickest, followed by the ring plasmid (II) and the line plasmid (III) [28]. Line structure was developed and superhelix plasmids dissociated completely when ZNF191(243-368)-His_8_ was added. This indicated that coordination of His and zinc ions endowed the protein with DNA pyrolysis. This may explain why H_4_-zinc finger structures do not exist in living body. But this is not available in His_6_-ZNF191(243-368), which further confirmed that different positions of polyhistidine will have different effects. Polyhistidine at the C-terminal end of the zinc finger protein may be easier to form activity site similar in protease [29].

## 4. Conclusions

His-tags at the N-terminal end and C-terminal end of ZNF191(243-368) have different influences on the properties and structure of proteins. Therefore, it is necessary to consider carefully whether to choose His-tag system for* in vitro* expression of zinc finger proteins. ZNF191(243-368) with His-tag had different properties, which provided new insights into the design of biological functional molecules.
